# Digestibility of a Lignocellulose Supplemented Diet and Fecal Quality in Beagle Dogs

**DOI:** 10.3390/ani12151965

**Published:** 2022-08-02

**Authors:** Amr Abd El-Wahab, Bussarakam Chuppava, Diana-Christin Siebert, Christian Visscher, Josef Kamphues

**Affiliations:** 1 Institute for Animal Nutrition, University of Veterinary Medicine Hannover, Foundation, Bischofsholer Damm 15, D-30173 Hannover, Germany; amrwahab5@mans.edu.eg (A.A.E.-W.); sekretariat-tierernaehrung@tiho-hannover.de (D.-C.S.); christian.visscher@tiho-hannover.de (C.V.); josef.kamphues@tiho-hannover.de (J.K.); 2 Department of Nutrition and Nutritional Deficiency Diseases, Faculty of Veterinary Medicine, Mansoura University, Mansoura 35516, Egypt

**Keywords:** dog, lignocellulose, apparent nutrient digestibility, fecal consistency

## Abstract

**Simple Summary:**

Obesity is not only a serious health problem for humans, but is also recognized as an immense health threat in pets. The use of lignocellulose in extruded diet might be used in pet food companies as a strategy to reduce energy density. These findings suggest that including lignocellulose supplementation up to 4% in dogs’ diets was well accepted, with an allowable reduction of the apparent digestibility of the control diet. Moreover, the stool remains solid and well-formed , and it increases fecal dry matter, indicating that it should be considered as a potential source of dietary fiber in dog food.

**Abstract:**

Lignocellulose (LC) might be used as a substitute fiber source for dogs as a strategy to decrease energy density and enhance gastrointestinal functionality. The objective of the present study was to compare the effects of including different levels of LC on apparent nutrient digestibility and fecal parameters (dry matter (DM), fecal score, and daily fecal output), as well as fecal fatty acid concentrations. Four diets were tested: control diet (no supplementation of LC; LC0), and three control diets diluted with increasing levels of LC: 1, 2, and 4% (LC1, LC2, and LC4). Six Beagle dogs (BW 17.1 kg ± 1.22) participated in a crossover experimental design. Before each experimental period, five days were used as a wash-out period. The fecal consistency was scored based on a 5-point scale (1 = very hard; 2 = solid, well-formed “optimum”; 3 = soft, still formed; 4 = pasty, slushy; and 5 = watery diarrhea). The results demonstrated that the organic matter apparent digestibility (*p* = 0.01) and nitrogen-free extract (NFE) (*p* < 0.01) was significantly lower for dogs fed LC4 compared to those fed LC0. Dogs fed LC2 and LC4 had a lower fecal consistency score (2.39 and 2.18, respectively; *p* < 0.05). The fecal DM was significantly the highest (*p* < 0.01), and the daily fecal output on fresh matter was lower (*p* < 0.05) when dogs were fed the LC4 diet compared with the LC0 diet. Including LC at 1% in the diet resulted in significantly higher fecal acidic pH levels. However, no differences among treatments were noted regarding fecal fatty acid concentrations.

## 1. Introduction

Dietary fiber is an increasing interest in pet nutrition regarding nutrient availability and gastrointestinal health and function [1,2,3,4,5]. Obesity is a common nutritional disease among pets and humans [6,7]. In 2017, the Association for Pet Obesity Prevention (APOP) [8] found that 60% of cats (33.5% obese/26.5% overweight) and 56% of dogs were obese or overweight (19.6%/36.4%, respectively). Generally, obesity in dogs and humans is not caused by a disease of an individual, but rather socioeconomic and environmental factors have influence on it and it derives from the close relationship between the dog and the owner [9]. It is well known that obesity could worsen the quality of life for both humans and dogs, as well as shorten the lifespan [7,10]. Moreover, obesity can induce or aggravate the body systems, such as respiratory, orthopedic, metabolic, endocrinal, oncologic, and cardiovascular disorders in humans and dogs [11,12]. Therefore, weight loss is a key factor for improving the quality of life and overall longevity. Moreover, dietary fiber is fermented by gut microorganisms, having been associated with gut health and the extension of fermentation, though it is affected by its structure [13].

Pet food companies produce diets with reduced calorie density and/or energy content by reducing fat and supplementing with fibrous ingredients that are aimed to reduce body weight and help with weight management [2,3,5,14]. The animal’s digestive enzymes cannot digest fiber, unlike starch in the diet; for this reason, it contributes few, if any, calories. In the past, cellulose has been a common source of fiber in low-calorie diets [3,15,16]. Previous studies have demonstrated that poorly fermented cellulose has a moderate capacity to bind water and can decrease the transit time of food and digestibility [17], as well as increase fecal output in dogs [18,19,20]. However, cellulose is expensive when compared to other fiber sources [5,14]. Thus, there is a need to find alternative fiber sources.

Lignocellulose (LC) is well recognized for the manufacturing of biofuels and as a source of renewable energy [21,22], but it could be used as a substitute for common fiber sources in dog food. Nevertheless, to our knowledge, it has not yet been extensively studied in canines. The mechanical method of fibrillation is used to extract LC from fresh naturally dried wood [23]. Due to the physical structural differences among LC and cellulose, it is suggested that it has different effects on the intestinal tract. LC is comprised of cellulose as the main structure, with embedded hemicellulose, pectin, and lignin moieties [24,25]. According to these characteristics, it can be assumed that the fermentability of LC is low to moderate in the digestive tract of dogs [4].

The hypotheses were that LC would act differently compared to a control diet on digestibility and fecal characteristics in dogs. The aim of this study was to compare the effects of including different concentrations of LC on apparent nutrient digestibility, as well as fecal parameters (dry matter, fecal score, and daily fecal output).

## 2. Materials and Methods

The experiments were performed in accordance with German regulations and approved by the Ethics Committee of Lower Saxony for Care and Use of Laboratory Animals (LAVES, Niedersächsisches Landesamt für Verbraucherschutz und Lebensmittelsicherheit; reference: 33.12-42502-04-13/1209).

### 2.1. Experimental Design and Diets

The digestibility trial was performed at the Institute for Animal Nutrition, University of Veterinary Medicine Hannover, Foundation, and included six healthy intact female Beagles (age ranged from 6 to 13 years). At the beginning of the trial and after the treatments, dogs’ body weight (BW) was recorded and body condition score (BCS) was evaluated by the same investigator in accordance with the 9-point BCS system, as validated by Laflamme [26]. A crossover design was chosen for the trials. The trial started with the animals acclimating to the meal for five days, followed by five days of collecting feces to measure apparent digestibility of nutrients and fecal characteristics individually. Before each experimental period (adaptation and collection), five days were used as wash-out.

As a control diet, a commercial extruded dry diet (animonda petcare GmbH, Osnabrück, Germany) containing poultry meat, corn, rice, beet pulp, animal fats, chicken liver, fish oil, yeast, spinach (dried), whole egg (dried), grape seed flour (0.25%), mussel extracts (0.1%), and minerals was used. The chemical compositions of the control diet are presented in Table 1. The calculation of the metabolizable energy (ME) content of foods was based on their chemical composition, as recommended by the National Research Council (NRC) [27]. The energy content of diets was analyzed to allow calculation of daily food amount (300 g/day). The amount of food provided was calculated using a computation according to their daily energy requirements (0.5 MJ ME BW^0.75^/d), based on the predictive equations of maintenance energy requirements for adult dogs following the metabolic weight according to NRC guidelines [27]. The control diet (LC0) was supplemented with different levels of LC to obtain a further three diets with 1, 2, and 4% LC in the control diet (LC1, LC2, and LC4, respectively). One hour before feeding, 100 mL of tap water was added to the dry diet. The animals were fed once a day and had unrestricted access to water. The amount of food offered and refused after each mealtime was recorded to calculate food intake.

### 2.2. Chemical Analysis

The Association of German Agricultural Analytic and Research Institutes e.V. (VDLUFA) established techniques to assess the nutrients in the diets and fecal samples [28]. The dry matter (DM) content was determined by weighing about 50 g of each sample before and after they were dried in an oven at 103 °C for 12 h. The crude ash content was determined by weighing approximately 3 g of each sample, which was placed in the muffle furnace and burned at 600 °C for 6 h. The total nitrogen values were measured in an elemental analyzer using the Dumas incineration method (Vario Max CNS, Elementar Analysensysteme GmbH, Langenfeld, Germany). Afterwards, the crude protein was calculated by multiplying the nitrogen content by 6.25.

The acid digestion and the Soxhlet extraction technique were used to determine the crude fat content. Petroleum ether was used to extract the fat for 6 h in a standing flask, followed by drying in a Soxhlet extractor (FOSS, Hilleroed, Denmark) until a constant weight, and distilled off with a rotary evaporator (Rotavapor R114, Fa. Büschi, Switzerland).

To determine the crude fiber content, the diluted acidic and alkalic solutions and subsequent drying at 103 °C were used (Fibertec 2010 Hot Extractor, Foss, Analytical AB, Höganäs, Sweden). Crude fiber was derived by subtracting the crude fiber content from the sample weight before ashing.

In accordance with the Association of Official Analytical Chemists (AOAC) [29], the calcium content was evaluated using an atomic absorption spectrometer (Solaar M-Serie Atomic Absorption Spectometer, Thermo Elemental Ltd., Cambridge, England). According to Gericke and Kurmies [30], the vanadate molybdate method was used to photometrically measure the phosphorus concentration (UV-Visible Recording Spectrophotometer UV 162; Wavelength 356 nm, Shimadzu Corporation, Kyoto, Japan). When orthophosphates are added to a reaction mixture containing ammonium molybdate, ammonium vanadate, and nitric acid, a yellow compound is formed that may be detected colorimetrically.

The nitrogen-free extract (NFE) content was determined using the following formula: dry matter − (crude ash + crude protein + crude fat + crude fiber).

### 2.3. Scores for Food Intake and Apparent Digestibility

The “food intake assessment” (palatability and speed of food intake) was divided into three scores in accordance with Abd El-Wahab et al. [31,32]. Briefly, score 1 = lowest acceptance; score 2 = moderate acceptance; score 3 = maximum acceptance.

The apparent digestibility of nutrients was assessed using the entire fecal collection method [33]. During the collection period, fresh feces were collected from the concrete floor every day. After weighing, the content of DM of a subsample of 10% fresh feces per animal/d was determined. After that, the remaining fecal samples were kept at –20 °C. At the end of the trial, the 5-day fecal samples from each dog were thawed, mixed, and homogenized. By multiplying ((food-feces)/food) by 100, the apparent digestibility (percent) was calculated [34].

### 2.4. Quality of Feces and pH Value

The fecal output was measured every day. For five consecutive days of the collecting phase, the feces were collected in their entirety and individually every 30 min. According to Moxham [35], the fecal consistency was measured on a five-point scale (1 = very hard, 2 = solid, well-formed “optimum”, 3 = soft, still formed, 4 = pasty, slushy, and 5 = watery diarrhea). To verify the pH of fresh pooled feces, the samples were combined in a 1:5 ratio with distilled water, shaken, and left for 1 min at room temperature, followed by measuring with a pH meter (InLab^®^ Expert Pro, Mettler-Toledo International Inc., Columbus, OH, USA).

### 2.5. Fecal Short-Chain Fatty Acids

According to Abd El-Wahab et al. [31], on the last day of the collecting phase, fresh feces were taken from each animal to measure fatty acids. Briefly, 1 g of feces was combined in a 1:5 ratio with purified water, vigorously agitated, then centrifuged at 2683 relative centrifugal force (rcf) for 15 min (Megafuge 1.0, Heraeus Deutschland GmbH & Co. KG, Hanau, Germany). After centrifuging the sample, it was subjected to gas chromatography at 155 °C (610 Series, Unicam Chromatography GmbH & Co. KG, Kassel, Germany) using an internal standard (10 mL formic acid 89 percent and 0.1 mL 4-methylvaleric acid). The carrier gas was nitrogen (flow rate: 0.97 mL/min), and flame ionization detection was used as gas chromatography detection method.

### 2.6. Statistical Analysis

All the analyses were performed using SAS^®^ version 9.3 (SAS Institute, Inc., Cary, NC, USA). Mean values and the standard deviation (SD) of the mean were performed for all parameters, while BCS was represented as the median. Univariate procedure was used for checking the normal distribution. The data with normal distribution were determined for significant differences among treatments with the REGWQ test (Ryan–Einot–Gabriel–Welsch multi-range test). For the data with non-normal distribution, i.e., defecation frequency or values in the form of a score, i.e., BCS-score, fecal consistency score, scores for food intake, the Kruskal–Wallis test was performed. Statistical level of significance was set at *p* < 0.05.

## 3. Results

### 3.1. Chemical Composition of Experimental Diets

Table 2 shows the chemical composition of the experimental diets. The DM content among the control diet and experimental diet was virtually the same (923 g/kg as fed). The contents of crude ash and crude protein, as well as ether extract, were decreased with an increasing inclusion level of LC, whereas the level of crude fiber increased with an increasing inclusion level of LC.

### 3.2. Apparent Fecal Nutrient Digestibility and Body Condition

The general condition of the dogs was healthy throughout the trial. No significant differences were noted in a mean BW between days and dietary groups (*p* > 0.05) at the beginning of the study (range: 17.3–17.5 kg; *p* = 0.141, Table 3), and remained constant throughout the experimental period (range: 17.4–17.6 kg; *p* = 0.709). When evaluating the BCS during the experimental period, no significant differences were observed between before and after the treatments among groups (*p* > 0.05, Table 3). The median BCS after the treatments was evaluated to be 5.0 out of the 9-point BCS system. Regarding food intake scoring, there were no significant differences among diet groups and the foods were well accepted; no refusals were observed during all the trials.

Table 3 shows the apparent digestibility of nutrients results. Dogs fed LC diets showed significant differences in organic matter (OM) and NFE digestibility. Apparent OM and NFE digestibility decreased with the addition of LC. OM digestibility was significantly higher for dogs fed LC0 compared to dogs fed the 2% and 4% LC supplemented diet (83.4% vs. 81.2% and 80.0%, respectively; *p* = 0.011). The control diet and LC2 showed a high NFE digestibility of 86.3% and 85.5%, respectively, while the 4% LC supplemented diet led to a lower digestibility (82.2%; *p* = 0.009). There were no effects of LC levels on crude protein digestibility (range: 76.3–77.1%; *p* = 0.626), crude fat (range: 93.6–94.4%; *p* = 0.709), and crude fiber (range: 11.1–19.5%; *p* = 0.403).

### 3.3. Fecal Output, Fecal Consistency, Fecal DM, and Fecal pH

The data on fecal quality are presented in Table 4. No significant differences were observed in daily defecation frequency (range: 1.90–2.23 time/day; *p* = 0.335). In contrast, dogs fed LC4 had markedly increased fresh fecal output compared to when they were fed the control diet (68.0 ± 3.98 vs. 57.0 ± 3.78 g DM/d; *p* = 0.037). When compared to dogs fed LC0, those fed LC4 had a lower mean fecal score (2.69 vs. 2.18; *p* = 0.047) with the highest fecal DM content (28.0% vs. 29.1%; *p* = 0.024). Additionally, the fecal pH level was different among treatment groups. The lower pH was measured for LC0 and LC2 (*p* < 0.001).

### 3.4. Fecal Short-Chain Fatty Acid (SCFA)

The results of the fecal fatty acid pattern are presented in Table 5. It was observed that there was no significant effect on the fecal fatty acid concentrations of dogs fed different levels of LC diets.

## 4. Discussion

In our study, LC2 and LC4 had significantly lower OM digestibility compared to LC0; however, only LC4 had significantly lower NFE digestibility compared to LC0 and LC2. Many factors can affect the diet digestibility, as well as nutrient quality and chemical composition [31,36,37]. Regarding the fiber content, Bednar et al. [38] and Kienzle et al. [39] found that digestibility of OM was lower for the diet containing a higher level of dietary fiber, which could possibly reduce nutrient digestibility. This is in accordance with our findings that lower OM digestibility was observed when feeding the diets with 2% and 4% LC compared to control diet (−2.2% and −3.4%, respectively). The crude fiber content of LC2 and LC4 was higher than the control diet (35.7 and 46.9 g/kg DM vs. 24.5 g/kg DM, respectively). The low values for apparent OM digestibility in the high-fiber diet are supposed to be due to the indigestibility of the fiber; thus, less material was available for the microorganisms to ferment, which led to a decreasing transit time and reduction in its absorption through the gastrointestinal tract [16,40]. However, in this study, there was no negative effect on protein digestibility when increasing levels of LC in the diet up to 4%.

Given the lower apparent digestibility of NFE when feeding the diets with 4% LC compared to the diet containing 0% of LC, it can also be assumed that the different crude fiber composition of the diet might be a reason for our results. Similarly, Kienzle et al. [41] found that the apparent NFE digestibility was decreased (from 93.4% to 85.9%) when dogs were offered diets with a higher fiber content (208 g/kg DM). The impact of fiber on the NFE digestibility was also in agreement with the results of Kienzle et al. [41], who stated that microbial degradation in the gut can transform some of the fiber into NFE. The high fiber content is difficult to ferment, leading to a shorter intestinal passage time, resulting in a reduction in bacterial carbohydrate fermentation in the colon [5]. This, in turn, results in a decrease in the apparent NFE digestibility and an increase in the apparent digestibility of fiber [41]. However, as found in the present study, no negative effect on apparent digestibility of crude fiber was observed when compared to those dogs fed the control diet. The current findings suggested that dietary supplementation with 4% LC had no negative effect on body condition despite low OM and NFE digestibility. However, the effect of replacing the dietary fiber sources with LC for weight loss and management on BW and BCS changes should be shown with long-term studies [42,43]. In the high fiber canine weight loss foods study of Yamka et al. [42], a significant change was found after 1 month of trial.

Differences in digestibility in the diets are also correlated with differences in fecal characteristics [40,44]. Other studies have shown that the frequency of defecation increases when dietary fiber was supplemented in the diet [45,46]. However, in our study, no significant differences were observed in daily defecation frequency between the groups fed a diet with or without a LC supplementation (range: 1.90–2.23 time/day). This is in contrast to dogs fed a diet supplanted with 4% LC, which had markedly increased fecal output compared with when they were fed the control diet. Low digestible diets result in a high fecal output and firm fecal consistency, characteristics that are of interest to pet owners [47]. Fahey et al. [48] noted that dogs consuming high dietary fiber had a higher fecal output as the percentage of dietary fiber increased.

Overall, fecal quality can be determined using either fecal consistency scores or DM content in the feces [43,47]. The fecal consistency scores can be influenced by a variety of factors, such as undigested total dietary fiber content and proteins [38,49]. In this study, increasing concentrations of LC in the diet resulted in fecal consistency scores closer to the optimal value (score 2), with greater fecal DM. Scores of fecal consistency remained at acceptable levels, with a range of 2.18–2.69. Thus, under the influence of LC supplementation (up to 4%) under these experimental conditions, the fecal quality could be demonstrated. This agrees with previous studies by Fahey et al. [48] and Kröger et al. [4], who found an influence of the type and amount of fiber source on the fecal quality. The dietary factors affecting fecal DM content, such as fiber digestion and absorption, as well as fiber fermentation activity, should be considered [49,50,51].The concentrations of fecal short-chain fatty acid (SCFA) have been used in practice to assess the activity of the large gut microbiota fermentation of dogs [52,53]. SCFA are particularly produced by microbial fermentation of undigestible carbohydrates [54], and the proportions of the SCFA may change depending on the fiber sources [47,55,56,57]. Our results showed that including LC in the diet had no negative effects for the dogs regarding the production of SCFA, although LC consists of high fibrous ingredients. However, McNeil et al. [58] and Harris et al. [59] reported that up to 95% of the SCFA produced are rapidly absorbed by the colon, and that fecal analysis may not be the best response criterion reflecting the host animal’s SCFA status. In addition, the results of the fecal fatty acid pattern in this study are determined from dogs fed diets supplemented with LC over a short period of time. Future studies with a longer study period might give further information on gut microbiota.

## 5. Conclusions

Lignocellulose offers some advantages compared with other fiber sources. Our study shows that including LC up to 4% in the dogs’ diets was well accepted, with dogs consuming the total amount of the food offered. Furthermore, it promotes well-formed feces, solid in consistency, and increases fecal dry matter compared with feces from dogs fed the LC0, with acceptable reduction of the apparent digestibility of the control diet. This suggests that it should be considered as a potential dietary fiber source in dog food.

However, this study had a limitation: the LC supplemented diet was fed over a relatively short period of time. Therefore, further studies conducted over a long-term period are still needed to investigate the effect of replacing dietary fiber sources with LC for weight loss and management, as well as on the plasma metabolites, fecal microbiota, and the changes in SFCA of dogs. Future investigation such as the effect of particle size on LC digestibility is still needed. A variety of causes can be considered; not only feeding management, but also genetics, age, and sex. Thus, in the future, it would therefore be interesting to test other breeds, such as Pugs and Golden Retrievers that are known to be at risk of developing obesity problems.

## Figures and Tables

**Table 1:** Chemical composition of the control diet.

Parameter	Unit	Control Diet
DM	g/kg as fed	923
Crude ash	g/kg DM	70.4
Crude protein		302
Ether extract		140
Crude fiber		24.5
NFE		386
Calcium		16.2
Phosphorus		10.8
ME	MJ/100 g DM	12.1

DM = dry matter, NFE = nitrogen-free extract, ME = metabolizable energy.

**Table 2:** Chemical composition of the ingredient and control diets supplemented due to different lignocellulose (LC) levels.

Item	Unit	Ingredient	Experimental Diets			
		Lignocellulose	LC0	LC1	LC2	LC4
Control diet	%	-	100	99	98	96
LC			0	1	2	4
DM	g/kg as fed	913	923	922.9	922.8	922.6
Crude ash	g/kg DM	43.8	70.4	70.1	69.9	69.3
Crude protein		16.5	302	299	296	291
Ether extract		6.24	140	139	137	135
Crude fiber		585	24.5	30.1	35.7	46.9
NFE		262	386	385	384	381
Calcium		4.32	16.2	16.1	16.0	15.7
Phosphorus		0.33	10.8	10.7	10.6	10.4
ME	MJ/100 g DM	4.69	12.1	12.0	11.9	11.8

Sum of crude ash, crude fat, crude protein, crude fiber, and N-free extracts (NFE) may not total 1000 g due to rounding up. Metabolizable energy (ME) content of foods were estimated based on their chemical composition, as recommended by the NRC [27].

**Table 3:** Apparent digestibility of nutrients (%), and body condition of dogs fed control diets supplemented with different levels of LC (mean ± SD).

Parameters	LC0	LC1	LC2	LC4	*p*-Value
Organic matter	83.4 ^a^ ± 1.13	81.9 ^ab^ ± 2.42	81.2 ^b^ ± 0.97	80.0 ^b^ ± 1.29	0.011
Crude protein	77.1 ± 1.28	76.5 ± 2.82	76.3 ± 1.72	77.1 ± 1.73	0.626
Crude fiber	19.5 ± 9.58	17.8 ± 11.5	11.1 ± 5.71	15.7 ± 7.33	0.403
Crude fat	94.2 ± 1.87	93.6 ± 1.18	93.8 ± 1.09	94.4 ± 0.46	0.709
NFE	86.3 ^a^ ± 1.56	84.4 ^ab^ ± 3.33	85.5 ^a ±^ 1.78	82.2 ^b^ ± 1.39	0.009
BW at start, kg	17.4 ± 1.46	17.5 ± 1.54	17.3 ± 1.21	17.3 ± 1.50	0.141
BW at final, kg	17.4 ± 1.38	17.6 ± 1.67	17.5 ± 1.35	17.5 ± 1.50	0.709
BCS * at start	5.0 (4.0–5.0)	5.0 (4.0–5.0)	4.5 (4.0–5.0)	4.5 (4.0–5.0)	0.192
BCS * at final	5.0 (4.0–5.0)	5.0 (4.0–5.0)	5.0 (4.0–5.0)	5.0 (4.0–5.0)	0.216

LC = lignocellulose. NFE = nitrogen-free extract. ^a,b^ Means in a row with different superscripts differ significantly (*p* < 0.05). * Body condition score (BCS) are represented as the median (range).

**Table 4:** Fecal characteristics of dogs fed the control diets supplemented with different levels of LC (mean ± SD).

Parameters	LC0	LC1	LC2	LC4	*p*-Value
Fecal consistency score (1–5) ^1^	2.69 ^a^ ± 0.60	2.54 ^ab^ ± 0.53	2.39 ^b^ ± 0.55	2.18 ^b^ ± 0.39	0.047
Fecal DM (%)	28.0 ^b^ ± 2.00	28.4 ^b^ ± 2.61	28.6 ^b^ ± 2.00	29.1 ^a^ ± 1.96	0.024
Fecal output (g DM/d)	57.0 ^b^ ± 3.78	62.0 ^ab^ ± 7.76	65.0 ^ab^ ± 3.12	68.0 ^a^ ± 3.98	0.037
Fecal pH	6.39 ^c^ ± 0.42	6.87 ^a^ ± 0.34	6.42 ^c^ ± 0.39	6.66 ^b^ ± 0.37	<0.001

LC = lignocellulose. ^a,b,c^ Means in a row with different superscripts differ significantly (*p* < 0.05). ^1^ Fecal scores were recorded using a five-point scale (1 = very hard, to 5 = watery diarrhea) and a score of 2 was considered optimal.

**Table 5:** Fatty acid concentration in the feces (mmol/kg fresh feces) of dogs fed the control diets supplemented with different levels of LC (mean ± SD).

Parameters	LC0	LC1	LC2	LC4	*p*-Value
acetic acid	61.1 ± 2.17	62.8 ± 3.49	63.3 ± 3.39	63.1 ± 3.20	0.812
propionic acid	19.3 ± 4.31	18.3 ± 5.35	21.7 ± 2.62	20.3 ± 4.01	0.899
iso-butyric acid	1.40 ± 0.32	1.10 ± 0.42	1.50 ± 0.29	1.40 ± 0.37	0.555
n-butyric acid	16.1 ± 5.09	16.0 ± 5.41	11.5 ± 1.77	13.3 ± 4.25	0.121
iso-valeric acid	2.10 ± 0.42	1.60 ± 0.24	2.00 ± 0.32	1.80 ± 0.47	0.197
n-valeric acid	0.00 ± 0.00	0.10 ± 0.20	0.00 ± 0.00	0.10 ± 0.10	0.218

LC = lignocellulose.

## Data Availability

The data presented in this study are available in this manuscript.
